# 3D Printed Ultrastretchable, Hyper-Antifreezing Conductive Hydrogel for Sensitive Motion and Electrophysiological Signal Monitoring

**DOI:** 10.34133/2020/1426078

**Published:** 2020-12-02

**Authors:** Zhaolong Wang, Lei Chen, Yiqin Chen, Peng Liu, Huigao Duan, Ping Cheng

**Affiliations:** ^1^National Research Center for High-Efficiency Grinding, College of Mechanical and Vehicle Engineering, Hunan University, Changsha 410082, China; ^2^MOE Key Laboratory for Power Machinery and Engineering, School of Mechanical and Power Engineering, Shanghai Jiao Tong University, Shanghai 200240, China

## Abstract

Conductive hydrogels with high stretchability can extend their applications as a flexible electrode in electronics, biomedicine, human-machine interfaces, and sensors. However, their time-consuming fabrication and narrow ranges of working temperature and working voltage severely limit their further potential applications. Herein, a conductive nanocomposite network hydrogel fabricated by projection microstereolithography (P*μ*SL) based 3D printing is proposed, enabling fast fabrication ability with high precision. The 3D printed hydrogels exhibit ultra-stretchability (2500%), hyper-antifreezing (-125°C), extremely low working voltage (<100 *μ*V), and super cyclic tensile stability (1 million cycles). The hydrogel-based strain sensor can probe both large-scale and tiny human motions, even with ultralow voltage of 100 *μ*V at extremely low temperature around −115°C. It is demonstrated that the present hydrogels can be used as a flexible electrode for capturing human electrophysiological signals (EOG and EEG), where the alpha and beta waves from the brain can be recorded precisely. Therefore, the present hydrogels will pave the way for the development of next-generation intelligent electronics, especially for those working under extremely low-temperature environments.

## 1. Introduction

The hydrogel is a group of polymeric materials with water held by its three-dimensional (3D) networks, which was firstly proposed by Wichterle and Lim in 1960 [1]. Different hydrogels, such as homopolymeric hydrogels, copolymeric hydrogels, and multipolymer interpenetrating polymeric hydrogels, have been prepared based on either chemical or physical nature of the cross-link junctions afterwards [2]. The merits of hydrogels with high stretchability and conductivity greatly extend their applications [3, 4] in electronics [5–7], biomedicine [8–10], power sources [11, 12], robots [13], actuators [14], human-machine interfaces [15], as well as sensors [16]. A stretchable conductive hydrogel conducts electricity by using either ions or electrons in a network including carbon-based materials, metal nanowires, liquid metals, conductive polymers, and ionic salts [3, 4, 17, 18]. Therefore, salts, such as NaCl and LiCl, are always used in the fabrication of the conductive hydrogel [8, 19], while polyacrylamide (pAAm) [8], polyvinyl alcohol (PVA) [20], and poly(2-acrylamido-2-methylpropane sulfonic acid) (PAMPS) are usually chosen as the network for their high stretchability and easy synthetization [18]. In addition, poly(ethylene glycol) diacrylate (PEGDA) is employed to shape the hydrogel because it always acts as a cross-linker [21]. Besides, nanoparticles are also used to enhance the stretchability of hydrogels [22, 23]. For instance, Odent et al. [22] designed and fabricated a stretchable, tough, conductive hydrogel by using dynamic and reversible interactions between a polymer network and silica nanoparticles.

Recently, the adaptability of the hydrogel to low-temperature environments has received much attention. With the addition of the ethylene glycol, it has been shown that the freezing point of the hydrogel can be lowered considerably and a specially designed hydrogel works well at a temperature of -35°C [24, 25]. An ionic liquid (IL) based ionogel can further lower its working temperature to -75°C [21]. However, it should be noted that most of the hydrogels are manufactured by chemical reactions, which usually need tens of hours for manufacturing a sample [21], and the working voltage is always higher than 0.1 V for those devices made of hydrogels [4]. Also, shaping the hydrogel and enhancing the stretchability [26] are always in conflict with each other. Therefore, how to further enhance the performance of the hydrogel needs to be investigated, and new processing methods should be developed.

Additive manufacturing of complex 3D micro- and nanoscale structures has attracted considerable attentions in the past three decades [27]. In the past five years, 3D printing technology promises the fabrication of complex hydrogel geometry constructs, which enables new functionalities together with improved performance [28, 29]. Indeed, nozzle-based direct ink writing was employed for constructing 3D structured hydrogels. Hydrogel precursor solutions including natural polymers such as agar, gelatin, chitosan, and synthetic polymers like acrylamide and N-isopropylacrylamide have been used in such a processing method [30]. However, poor printing resolutions (hundred microns) of the equipment [31] and mechanical robustness of the printed hydrogel [17, 32] severely limit the applications of the nozzle-based direct ink writing technique for hydrogel fabrication. Alternatively, a photopolymerization-based 3D printing technique is attracting more and more attentions in hydrogel fabrication because of its high precision with l-hydroxy-cyclohexyl-phenyl-ketone, ethyl (2,4,6-trimethylbenzoyl) phenylphosphinate (TPO-L), 2-hydroxy-2-methylpropiophenone, or 2,2-dimethoxy-2-phenylacetophenone acting as a photoinitiator [22, 33, 34]. However, compromises need to be made for the printing process because of the fact that most of these hydrogel structures exhibit mechanical brittleness and low stretchability. The obvious defects of these previously printed hydrogels by using the photopolymerization-based 3D printing technique also restrict their applications. Nevertheless, it should be noted that 3D printing techniques can fabricate an extremely complex 3D structure within a few minutes, which is totally impossible by using any other processing methods. Therefore, it is necessary and meaningful to further develop conductive hydrogels with excellent stability and ultrastretchability by using a high-precision 3D printing system for specific applications.

In the present study, we propose a new type of hydrogel which combines advantages of high stretchability, high conductivity, as well as ultralow working voltage and freezing point. The hydrogel is fabricated by the P*μ*SL based 3D printing technique, enabling greatly shortened fabrication time down to a few seconds for each layer while with high precision. Moreover, these hydrogels are used as flexible and wearable strain sensors to probe human activities of both large-scale and tiny motions even with an ultralow working voltage of 100 *μ*V at an extremely low temperature around −115°C. These hydrogels also are tested as a flexible electrode for capturing human electrophysiological signals (EOG and EEG), where the alpha and beta waves from the brain can be recorded precisely.

## 2. Results and Discussion

### 2.1. Fabrication and Characterization of the Hydrogel

**Figure 1:**
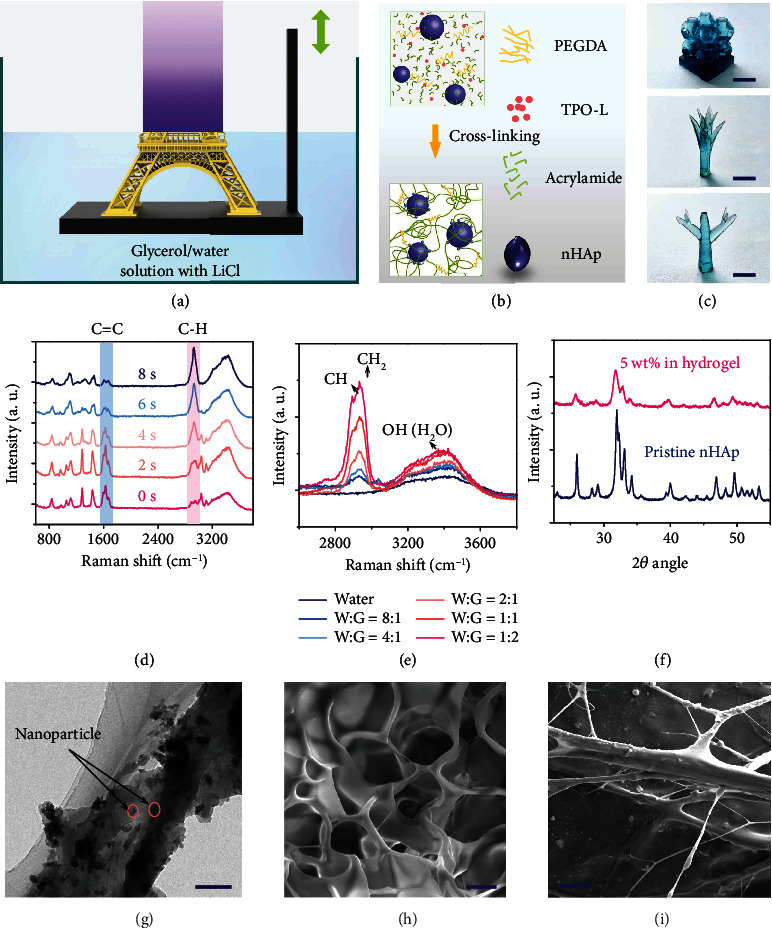
Preparation and characteristics of the hydrogel. (a) Schematic of the high-resolution fast up-bottom fabrication of the P*μ*SL technique. (b) Structural characterization of hydrogels via photopolymerization in the presence of nanoparticles. (c) Photographs of the complex structures made of the proposed hydrogel, including the Kelvin foam and tree-like complex 3D structures (dyed with methylene blue); the scale bar is 5 mm. (d) Raman spectra of the C=C and C-H in the hydrogel. (e) Raman spectra of the CH, CH_2_, and O-H in the hydrogel with different weight ratios of water/glycerol. (f) XRD spectra of the distribution of the hydroxyapatite nanoparticles. (g) TEM of the printed conductive hydrogel; the scale bar is 200 nm. (h) SEM of the printed conductive hydrogel without nanoparticles; the scale bar is 5 *μ*m. (i) SEM of the printed conductive hydrogel with nHAp; the scale bar is 10 *μ*m.

The conductive hydrogel structures were fabricated by using the P*μ*SL technique (Figure 1(a)). A LED light source of 405 nm was used for the solidification of the photocurable polymer solution. An elaborative precursor solution, consisting of AAm, LiCl, nHAp, PEGDA, and TPO-L solvated by glycerol/water with low viscosity, was prepared to meet the requirements of high-resolution features and fast fabrication by the 3D printing system (Figure 1(b), Figure S1). In the hydrogel network (Figure 1(b)), the PEGDA segments act as “hard domains,” which are the backbone of the hydrogel architectures and determine the printing precision. In contrast, the AAm segments serving as “soft domains” are mainly responsible for the high stretchability of the hydrogel [35]. TPO-L is a photoinitiator, and glycerol is an antifreezing solvent, which enables the free radical photopolymerization of AAm and PEGDA cross-linkers with 405 nm light. It is worth noting that TPO-L has been commonly used as a photoinitiator for its excellent absorbing characteristics in the deep blue to near UV [36]. However, TPO-L is almost insoluble in water, which seriously limits its application as photopolymerization for previous hydrogel printing.

The key to our fast fabrication is the addition of TPO-L to glycerol. Then, the solution was added to water followed by an ultrasonic bath to obtain uniform emulsion, in which TPO-L was solubilized completely and thus guaranteed the UV curing and speed of fabrication. Moreover, the glycerol not only was used as the solvent for TPO-L but also acted as an antifreezing component. Similarly, LiCl dissolved in water and glycerol solution acted as an ionic conductor, and it also contributed to freezing point depression with glycerol synergistically, though the kinematic viscosity of hydrogel precursor solution increased with the increasing concentration of LiCl (Figure S2). In addition to the mechanical reinforcement afforded by the presence of the AAm soft domain, nHAp (Figure S3) was also added to further enhance the tensile stretchability and stress of the hydrogel (Figure 1(b)). The samples demonstrate the ability to fabricate complex 3D structures by using the precursor solution with the P*μ*SL technique, including the Kelvin foam model and another two tree-like complex 3D structures with sharp tips (Figure 1(c)) [26].

By using Raman spectroscopy to detect changes in chemical bonds during polymerization from a liquid state to a solid state, the signal of C-H becomes stronger with the decrease of the C=C signal within 8 seconds (Figure 1(d), Table S1), indicating that the curing time from liquid solution to a solid layer is within 8 s by the P*μ*SL technique, which is far quicker than those of traditional methods [4, 37]. The peak attributed to glycerol observed from Raman spectroscopy increases with the increasing glycerol/water ratio, and the peak of the O-H at 3450 cm^−1^ in water moves toward smaller wavenumbers, indicating that these groups are strongly affected by hydrogen bond (Figure 1(e)). The addition of nHAp can be revealed by the appearance of a peak on X-ray diffraction (XRD) spectrum (Figure 1(f)), which corresponds to the phosphate group on nHAp. The transmission electron microscope (TEM) image (Figure 1(g)) and energy-disperse spectroscopy (EDS) result (Figure S4) of the hydrogel validate the uniform distribution of nHAp in the hydrogel.

It should be noted that microstructures of hydrogels with and without nanoparticles are different. The scanning electron microscope (SEM) images of the proposed 3D printed hydrogel with and without nanoparticles were captured (Figures 1(h) and 1(i)). A comparison of the two figures shows that the structure of the hydrogel changes from the one with thin walls to microfibers. The change of microstructures within the hydrogel is supposed to come from the physical connection between nHAp and covalent cross-linked polymer chains including pAAm as soft chains as well as PEGDA as hard chains. The fibrillation of the hydrogel results in unique mechanical properties with excellent elongation for the proposed hydrogel.

### 2.2. Stretchability and Freezing Resistance of the Hydrogel

**Figure 2:**
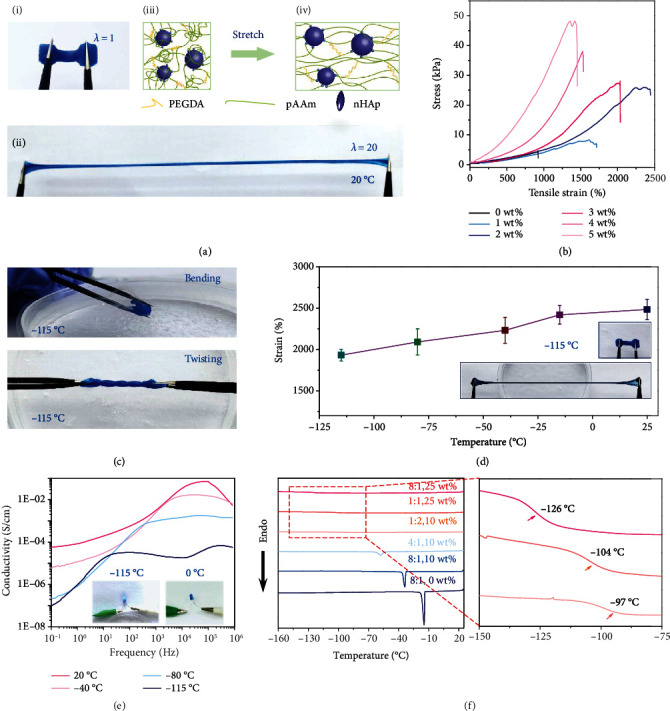
Mechanical and antifreezing properties of the proposed hydrogel. (a) Photographs demonstrating the stretchability of the hydrogel at normal temperature: (i) the proposed hydrogel, (ii) a stretched hydrogel for 20 times of its original length, (iii) the schematic of the microstructure of the proposed hydrogel, and (iv) the schematic of the microstructure of a stretched hydrogel. (b) Tunable tensile strain of the proposed hydrogels with different weight concentrations of nHAp. (c) Tortuosity of the proposed hydrogel at -115°C. (d) The effect of the temperature on the stretchability of the proposed hydrogel. (e) The effect of the temperature on the conductivity of the proposed hydrogel: a LED lamp test is inserted in the figure to show the comparison of the conductivity of the proposed hydrogel working at -115°C (left) and a previously reported one working at 0°C (right). (f) DSC measurement of the proposed hydrogel with different components.

The stretchability of the proposed hydrogel is schematically illustrated in Figure 2(a). It can be seen that the proposed hydrogel can easily be stretched to 20 times of its original length at 20°C (Figures 2(a-i) and 2(a-ii)). The assumable underlying mechanism responsible for the excellent stretchability is revealed (Figures 2(a-iii) and 2(a-iv)). Covalent cross-linked polymer chains including pAAm and PEGDA show a dynamic adsorption/desorption on surfaces of nHAp because of the physical connection and interaction between nHAp and polymer chains (Figure 1(i)). When the hydrogel is highly stretched, the dynamic adsorption/desorption works at high deformations and rearranges the physically connection, as well as the interaction between pAAm, PEGDA chains, and nHAp [23, 38]. This rearrangement compensates for rupture of covalent bonds in cross-linked polymer chains at high deformations, extending the elongation of the hydrogel to the maximum. The mechanical properties of the proposed hydrogel with different concentrations of nHAp are also studied (Figure 2(b)). It can be seen that the hydrogel can be stretched at most twenty-five times to its original length, which is far beyond the performance of the hydrogel without nHAp (Figure S5). More significantly, the stretchability of the hydrogel increases with the increasing concentration of nHAp before reaching the maximum around 2 wt%, and then, the stretchability decreases with further increasing the concentration of nanoparticles. However, it should be noted that the tensile stress of the proposed hydrogel increases with the increasing concentration of nanoparticles, and the balance of tensile stress and stretchability can be adjusted by the concentration of the nanoparticles. Most importantly, the viscoelasticity performance of the hydrogel from the relation of tensile strain indicates the dynamic adsorption/desorption between nHAp and covalent cross-linked polymer chains as well (Figure 2(b)) [23]. In addition, the weight ratio of PEGDA/AAm and the speed of drawing also strongly affects the mechanical behavior of the proposed hydrogel because the PEGDA domains the strength of the hydrogel and the speed of drawing greatly affects the physical interaction between nHAp and polymer chains (Figure S5).

Owing to the presence of glycerol and LiCl synergistically, hydrogels can be bended and twisted at the surface of liquid nitrogen (Figure 2(c)). The test of the proposed hydrogel was carried out between -115°C and 20°C (inserted figures of Figure 2(d)), and the temperature was controlled by adjusting the distance between the samples and the surface of liquid nitrogen (Movie S1). It is observed that the stretchability of the present hydrogel decreases with the decreasing of the temperature, but the hydrogel can still be stretched to 1800% of its original length at −115°C (Figure 2(d), Movies S1 and S2). The conductivity of the hydrogel at a subzero temperature was also investigated. The conductivity of the 3D printed conductive hydrogel decreases with the decrease of the surrounding temperature (Figure 2(e)). The lower the temperature, the slower the motion of the ions, leading to the decrease of the electrical conductivity of the hydrogel. In addition, the conductivity performance of the proposed hydrogel and a previously reported one at respectively -115°C and 0°C is compared (right figure of Figure 2(e), Figure S6, Movies S3 and S4). It can be seen that the LED was lightened when connected by the present hydrogel at -115°C, while the LED did not work when connected by a previously reported hydrogel [23] for comparison even at 0°C. Besides, the high concentration of LiCl, the low weight ratio of PEGDA/AAm, and the high weight ratio of water/glycerol also increased the conductivity of the hydrogel (Figure S7).

Differential scanning calorimetry (DSC) was carried out from −160°C to 25°C to further investigate the freezing point of these 3D printed hydrogels (Figure 2(f)). For hydrogels without LiCl, a sharp peak of freezing point was observed at -15°C, which can be attributed to the ice crystals formed in the hydrogel. From the comparison of these three lines presenting freezing point or glass transition temperature of samples with weight ratios of water/glycerol of 8 : 1, 4 : 1, and 1 : 2 (10 wt% LiCl), it can be seen that the freezing point or glass transition temperature of hydrogels first drastically decreases with the increase of the glycerol and then increases with the increase of the glycerol after reaching its minimum with a weight ratio of water/glycerol of 8 : 1 (25 wt% LiCl). In addition, from the comparison of lines representing the samples with different concentrations of LiCl (the weight ratio of water/glycerol is 8 : 1), it turns out that the glass transition temperature of the hydrogel decreases with the increasing concentration of LiCl, indicating that ice crystals have been restrained. In fact, the movement of water molecules is restricted by the high concentration of salt and glycerol because of hydrogen bonds between glycerol and water as well as ion-solvent interactions [18, 25]. The salt and water/glycerol synergistically enhance antifreezing and conductivity properties of the present hydrogel because the coexistence of these components disrupts the formation of crystal lattices of ice at low temperatures, leading to the decreasing glass transition temperature of the hydrogel (Table S2). However, it should be noted that the conductivity of the proposed hydrogel decreases with the increase of the glycerol (Figure S7). The best case for the fabrication of the proposed hydrogel was set as follows with a glass transition temperature around −125°C (Figure 2(f)): the weight ratio of water/glycerol was 8 : 1 and the concentration of LiCl was 25 wt% in the entire paper unless otherwise indicated.

### 2.3. Human Motion Monitoring

**Figure 3:**
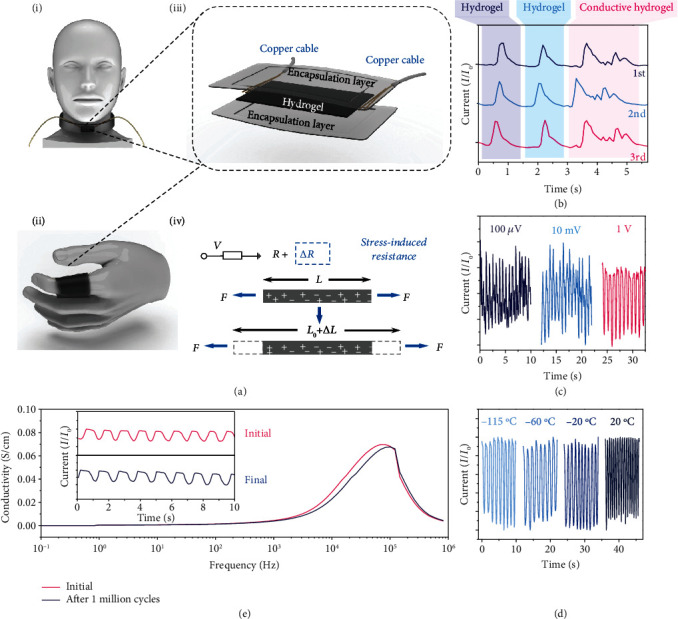
Performance of a flexible and wearable sensor made of the printed hydrogel. (a) Schematic illustration of the 3D strain sensor assembled from the conductive hydrogel: (i) detection of the muscle movements of the throat, (ii) the sensor used to monitor joint motions, (iii) the composition of the hydrogel sensor, and (iv) the underlying mechanism for the sensor. (b) The relative current changes versus time as a person pronounces the words “hydrogel, hydrogel, conductive hydrogel” for three times. (c) The signal of the sensor with different working voltages for detecting finger bending. (d) The signals of the sensor working at different temperatures by probing the bending of the finger. (e) The stability of the hydrogel sensor with 1 million cycles and the conductivity of the sensor before and after 1 million cycles were measured, and signals of finger motions before and after 1 million cycles were inserted.

The hydrogel had been used as a flexible strain sensor with high sensitivity for monitoring various human motions [39, 40]. The combination of ultrahigh stretchability and excellent conductivity of the present 3D printed hydrogel provides a versatile platform for sensing applications. The proposed hydrogel with electrodes and encapsulation layers was assembled for a flexible and wearable strain sensor (Figures 3(a-i)–3(a-iii)). The hydrogel was attached to two copper cables at two ends, while covered by two PDMS layers to avoid water escaping from the hydrogel. The resistance of the hydrogel changed with its length (Figure 3(a-iv), Figure S8), which can be used for the detection of motions. Theoretically, such a sensor can be utilized for probing almost all human motions due to the ultraflexibility and high sensitivity of the hydrogel, such as the motion of the fingers, wrist, elbow, ankle, and knee joints (Figure S8).

Of particular interest are some slight physiological motions, such as swallowing and vocal cord vibration. When the sensor was attached to the throat, the strain sensor directly and precisely monitored the subtle and complicated muscle movements of the throat when pronouncing different words (Figure 3(b)). The first two peaks on the curves of the relative current changes reflected the throat muscle motion when a person pronounced the same word of “hydrogel”. However, there is a distinct discrepancy between the signals of pronouncing “hydrogel” and “conductive hydrogel” when comparing the first two peaks with the third peak on the curves, validating the high sensitivity of the present hydrogel sensor. Furthermore, the three curves were almost the same when a person pronounced the phrase “hydrogel, hydrogel, conductive hydrogel” for three times, suggesting the good repeatability of the proposed hydrogel sensor (Figure 3(b)). Therefore, this hydrogel sensor can be potentially used as the detector for slight physiological motions, such as voice recognition and voice control switch for person unable to speak (Figure S8).

The hydrogel sensor was then fixed on the index finger by conductive tapes to monitor joint motions. The relative real-time current changes during the bending and stretching behaviors with different working voltages were recorded (Figure 3(c), Movie S5). The working voltage of those previous hydrogel electronics, including the sensors and ionotronic devices made of hydrogels, is always higher than 0.1 V [4], while the present 3D printed flexible and wearable strain hydrogel sensor could work with a constant voltage of 100 *μ*V. The possible electrochemical reaction during the functioning of ion conductors was eliminated effectively under such low voltage, suggesting the stability of the hydrogel as a strain sensor [41]. Signals from a hydrogel strain sensor of bending and stretching of a finger with a constant working voltage of 100 *μ*V were similar to those with a working voltage of 1 V and 10 mV, indicating an ultralow working voltage with three orders of magnitude lower than that of the existing hydrogels (Figure 3(c)). In addition, the performance of the sensor at −115°C, −60°C, −20°C, and 20°C was investigated (Figure 3(d)). The signals for these four temperatures are almost the same, demonstrating that the hyper-antifreezing property of the hydrogel enables the marvelous performance of the sensor at subzero temperatures. The excellent performance of the sensor tested at −115°C ensures great potential applications of wearable devices for human activity detection and health monitoring in cold environments (outside of protective clothing) and even vibration detection of aircrafts at high altitudes. To further evaluate the mechanical robustness and reliability of the hydrogel sensor, 1 million tensile cycles with 100% strain were applied (Figure 3(e), Movie S6). The highly elastic hydrogel at a small stain could recover to its initial length and still exhibited excellent sensitivity to probe human motions of bending and stretching a finger (inserted figure in Figure 3(e)) after 1 million cycles, indicating that the hydrogel sensor had excellent durability and stability in electromechanical behavior.

### 2.4. Human Neural Signal Capturing

**Figure 4:**
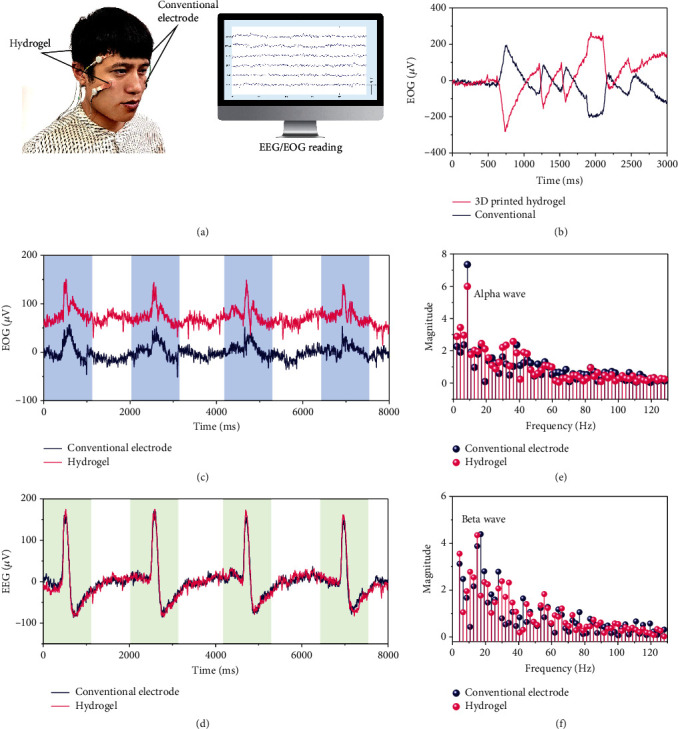
Performance of a flexible electrode made of the printed hydrogel for capturing human neural signals. (a) Illustration of the 3D printed flexible electrode acting as a human-machine interface. (b) The EOG of the nerve of horizontal rotation of the eye balls: the opposite signals indicate the EOG of right and left eyeballs. (c) The EOG signal of blinking the eyes. (d) The EEG signal of blinking the eyes. (e) The Fourier transformation of the signal of closing the eyes and relaxing. (f) The Fourier transformation of the signal of opening the eyes and focusing to show the neural activity.

The electroencephalogram (EEG) and the electrooculogram (EOG) are very important probes for neural signals in human beings. Enabled by the ultralow working voltage and conductivity of our proposed hydrogel, a flexible electrode for capturing EEG and EOG was fabricated, and neural activities were recorded by Compumedics E-Series Neurology Amplifiers & Recorders (Figure 4). The flexible electrodes made of the proposed hydrogel were attached to the left half of the head, while conventional electrodes were stuck to the right half of the head for comparison (Figure 4(a)). Interfacial impedance between hydrogels and the skin at different frequencies was firstly examined (Figure S9) because impedance values between the hydrogel and human skin should be below the threshold of 100 k*Ω* at a frequency of 1000 Hz to evaluate the availability of the electrodes [42]. The interfacial impedance of the present hydrogel flexible electrodes was far below the acceptable threshold of 100 k*Ω* at the required frequency around 10^3^ Hz, indicating the promising ability of the present hydrogel to record neural signals in the brain.

The EOG of horizontal rotation of the eyeball recorded by the proposed hydrogel electrodes and conventional ones synchronizes with each other, though the signals are in reverse with each other because the two signals are respectively for right and left eyeballs (Figure 4(b)). The EOG and EEG of blinking the eye are demonstrated in Figures 4(c) and 4(d). It turns out that the EEG from the proposed hydrogel and conventional electrodes are the same without much difference in both the signal shape and amplitude, and the peaks of the EOG from the hydrogel in the upper figure are even sharper than those recorded by the conventional electrodes. It can be seen from the Fourier transmissions of the EEG for closing the eyes and relaxing that the peak appears at 8 Hz, indicating the correct record of the alpha wave in the brain (Figure 4(e), Figure S9). In addition, the peaks from the Fourier transmissions of the EEG for opening the eyes and focusing demonstrate the change from alpha wave to beta wave in the brain after opening of the eyes (Figure 4(f), Figure S9). The performance of the present flexible electrodes demonstrates that they are one of the most promising candidates for human-machine interface with characteristics of easy fabrication (3D printing), high-resolution features for fabrication (Figure 1(c)), great conductivity, precise record of the electronic activities in nervous systems in the brain, and hyper-low freezing point (as low as -125°C). The hydrogel electrodes also have the potential to be used for mind-controlled activities in near-earth orbits at a temperature as low as -90°C.

## 3. Conclusions

In summary, we propose a conductive hydrogel by taking the advantage of the P*μ*SL based 3D printing technique for higher precision (<50 *μ*m) and shorter curing time for a layer (<10 s). The excellent characteristics of the hydrogel, such as ultrahigh stretchability (>2500%), hyper-antifreezing (-125°C), cyclic tensile stability (>1 million cycles), and good shaping property with tunable mechanical properties and tunable conductivity properties, come from a specific material portfolio of the hydrogel. The sensor made of the present hydrogel exhibits excellent sensitivity and stability to be used to probe human being's activities, including both large-scale and tiny motions in real time. In particular, the sensor works well under an extremely low voltage of 0.0001 V at a low temperature of −115°C. Most significantly, the hydrogel has attractive characteristics to be used as a flexible electrode. It is demonstrated that the flexible electrodes made of such a kind of hydrogel record the EEG and EOG precisely when rotating the eyeballs, and the performance of the flexible hydrogel electrodes is comparable with those of the conventional metallic electrodes, including recording alpha and beta waves in the brain. It is believed that the newly developed 3D printed hydrogel paves the way for the development of a new generation of intelligent electronics and bioelectronic interface, especially for those working under extremely low-temperature environments.

## 4. Materials and Methods

### 4.1. Materials

All chemicals were purchased from Sigma-Aldrich. Acrylamide (AAm), poly (ethylene glycol) diacrylate (PEGDA, Mw = 600Da), nHAp, glycerol, TPO-L, and LiCl, together with deionized water (18.2 M*Ω*·cm), were used to prepare the 3D printable hydrogel precursor solution while methylene blue was used to visualize the 3D printed hydrogel in photographs. All chemicals were of analytical grade and used without further purification.

### 4.2. Preparation of 3D Printable Hydrogel Precursor Solution

To synthesize the 3D printing hydrogel precursor solution, we prepared the AAm monomer solution (solution A) by adding AAm, LiCl, and nHAp to deionized water, while solution B was glycerol with PEGDA and TPO-L. After mixing solution A and solution B, the mixture was sonicated for 30 minutes. The weight ratios of PEGDA/AAm and water/glycerol were set to 0.01 : 2 and 8 : 1, respectively. In addition, the weight percentages of nHAp, photoinitiator TPO-L, and LiCl were respectively set to 2%, 1%, and 25% in the entire experiments unless otherwise indicated. Besides, we added methylene blue to the mixture which guarantees the high printing resolution of hydrogel microstructures and the visualization of hydrogels in photographs. Once finished, the precursor solution was well prepared for the following 3D printing after degassing for 10 minutes in the dark.

### 4.3. 3D Printing Fabrication

A P*μ*SL based 3D printer (S140, BMF) with 405 nm UV light was employed for the fabrication. We designed a solid 3D model by using SolidWorks and digitally sliced a 3D model into multiple layers with a 10-100 *μ*m thickness by using BMF slicing and control software for solidifying the hydrogel precursor solution layer by layer. The intensity of the LED light was 190 mW·cm^−2^, and the irradiation time for each layer was 8 s (Table S1). Once finished, we blew the printed structure with N_2_ to clear up the solution on the surface. For hydrogel structure with a high-resolution feature, the printed layer thickness was set to 10 *μ*m. For samples used for testing, the printed layer thickness was set to 50 or 100 *μ*m.

### 4.4. Material Characterization

The microstructures of hydrogels after removal of solvent were characterized by scanning electron microscopy (SEM, MIRA3-LMH, China). In addition, X-ray diffractometry (XRD, SmartLab-3kW, Japan), energy-disperse spectroscopy (EDS, X-MAX20, England), and transmission electron microscopy (TEM, Tecnai-G2-F20, USA) were used to demonstrate the uniform distribution of nanoparticles. Besides, a Raman spectrometer (Alpha300R, Germany) was used to indicate the photopolymerization process and group bonding. The freezing point or glass transition temperature of the printed hydrogels was measured by differential scanning calorimetry analysis (DSC8500, USA) from -160°C to 25°C.

### 4.5. Mechanical Measurement

Tensile measurements were carried out by using a tensile tester (ZQ-990LB, 20 N, China) with a constant stretching velocity of 10 mm·min^−1^. For tensile cyclic tests, a hydrogel was 200% stretched and then returned to its initial length with a constant frequency of 130 cycles per minute. The 1 million times cyclic test was performed continuously without interval between any two cycles.

### 4.6. Electrical Measurement

We obtained the conductivity of the hydrogels by a four-electrode alternating current (AC) impedance method over a frequency range of 0.1–10^6^ Hz by using the electrochemical workstation (CH1660H, Chenhua, China). The voltage of the electrochemical workstation during the test was set to 1 V. The conductivity is calculated from the formula *σ* = *Z*′/(*Z*^′2^ + *Z*^″2^) × *L*/*S*, where *σ* is the conductivity in S·cm^−1^, while *Z*′ and *Z*^″^ are the real and imaginary parts of the impedance, *S* is the cross section area of the hydrogel, and *L* is the hydrogel's thickness.

### 4.7. Assembling of a Strain Hydrogel Sensor

To make a strain sensor, we attached two individual copper electrodes to two sides of a printed hydrogel structure by conductive silver pastes. The above-mentioned system was then encapsulated with PDMS to prevent water evaporation from the hydrogel. Then, the hydrogel sensor was connected to a digital meter (Keithley 2611B) to reveal the performance of the sensor.

### 4.8. Recording of EEG/EOG Signals

To obtain the EEG/EOG signals, a simultaneous measurement system (E44, Compumedics E-Series) allowing parallel acquisition of EEG/EOG data from both commercial gold cup electrodes and our printed hydrogel electrodes with same size was employed. During an overall recording time, we recorded different EEG/EOG episodes including state EEG/EOG (eyes open), EEG/EOG with predominant alpha activity (eyes closed), and signals for eye blinking and horizontal rotation of the eyeballs.

## Supplementary Materials

Supplementary 1 Figure S1: preparation for the 3D printing of the hydrogel. Figure S2: the kinematic viscosity of hydrogel precursor solution with different concentrations of LiCl. Figure S3: SEMs for the hydrogels with different concentrations of nanoparticles. Figure S4: EDS maps and EDS spectra for the indication of the distribution of nanoparticles in the hydrogel. Figure S5: snapshots of stretching a hydrogel sample and tunable tensile strain of the proposed hydrogels with different stretching speeds and ratios of PEGDA/AAm. Figure S6: the performance of a LED bulb connected by hydrogels. Figure S7: conductivity of the printed hydrogel affected by different parameters. Figure S8: hydrogel sensor for detection of different physiological motions. Figure S9: neural interface for detecting different signals of EEG and EOG. Table S1: optimized parameters for 3D printing of the hydrogel using the S140 printer. Table S2: summary of results for stretchable and antifreezing gels.

Supplementary 2 Movie S1: flexibility and stretchability of the hydrogel at about −115°C.

Supplementary 3 Movie S2: ultrastretchable and antifreezing properties of the hydrogel.

Supplementary 4 Movie S3: conductive performance of the hydrogel.

Supplementary 5 Movie S4: conductive properties of the hydrogel at about −115°C.

Supplementary 6 Movie S5: hydrogel sensor for the detection of finger motion.

Supplementary 7 Movie S6: tensile cyclic tests of the hydrogel.
