# Supplementary material for: 3D Printed Ultrastretchable, Hyper-Antifreezing Conductive Hydrogel for Sensitive Motion and Electrophysiological Signal Monitoring
Source: Research (Wash D C). 2020 Dec 2;2020:1426078. doi: 10.34133/2020/1426078 (PMC7877384; doi:10.34133/2020/1426078)
Supplement: Supplementary 1 — Figure S1: preparation for the 3D printing of the hydrogel. Figure S2: the kinematic viscosity of hydrogel precursor solution with different concentrations of LiCl. Figure S3: SEMs for the hydrogels with different concentrations of nanoparticles. Figure S4: EDS maps and EDS spectra for the indication of the distribution of nanoparticles in the hydrogel. Figure S5: snapshots of stretching a hydrogel sample and tunable tensile strain of the proposed hydrogels with different stretching speeds and ratios of PEGDA/AAm. Figure S6: the performance of a LED bulb connected by hydrogels. Figure S7: conductivity of the printed hydrogel affected by different parameters. Figure S8: hydrogel sensor for detection of different physiological motions. Figure S9: neural interface for detecting different signals of EEG and EOG. Table S1: optimized parameters for 3D printing of the hydrogel using the S140 printer. Table S2: summary of results for stretchable and antifreezing gels. [file 1426078.f1.docx]

Supporting Information

**3D Printed Ultra-stretchable, Hyper-anti-freezing Conductive Hydrogel for Sensitive Motion and Electrophysiological Signal Monitoring**

*Zhaolong Wang*^†^*, Lei Chen*^†^*, Yiqin Chen, Peng Liu, Huigao Duan*, Ping Cheng**

*Corresponding authors. Email: [duanhg@hnu.edu.cn](mailto:duanhg@hnu.edu.cn) (H.D.); [pingcheng@sjtu.edu.cn](mailto:pingcheng@sjtu.edu.cn) (P.C.)

**Figure S1.** Preparation for the 3D printing of the hydrogel. (a) We prepared AAm monomer solution (solution A) by adding AAm, LiCl and nHAp to water. Solution B was the PEGDA solution, prepared by adding PEGDA and TPO-L to glycerol. After mixing solution A and solution B, the mixture was sonicated for 30 minutes. To reach high resolution for hydrogel microstructures manufactured by 3D printing technology, light absorber (methylene blue) was added to the mixture to obtain the precursor solution. The precursor solution was ready for 3D printing after mixing quickly and degassing for 10 minutes in the dark. (b) 3D printed high-resolution hydrogel grid.

**Figure S2.** The kinematic viscosity of hydrogel precursor solution with different concentration of LiCl. The kinematic viscosity of hydrogel precursor solution increases with the increase of the weight concentration of LiCl.

**Figure S3.** SEMs for the hydrogels with different concentrations of nanoparticles. (a) SEM images of the hydrogel without nanoparticle, which show highly porous structures with interconnected pores. (b) SEM images of the hydrogel with nanoparticle of 2 wt%, which have microfibers bonding structures with the addition of nanoparticle. (c) SEM images of the hydrogel with nanoparticle of 5 wt%, hydrogel-nanoparticle-aggregates were observed, the aggregation of the nHAp might occur during the process of 3D printing

**Figure S4.** EDS maps and EDS spectrum for the indication of the distribution of nHAp in the hydrogel. (a) SEM image of the hydrogel with nanoparticle of 2 wt%. (b) EDS maps of elements confirming the uniform distribution of nHAp and all other elements in the hydrogel. (c) EDS spectrum of nHAp in hydrogel.

**Figure S5.** Snapshots of stretching a hydrogel sample and tunable tensile strain of the proposed hydrogels with different stretching speeds and weight ratio of PEGDA / AAm. The tensile tests were carried out on a testing machine (ZQ-990LB, China) with a 20N load cell for tension. The tensile tests were performed at a speed of 10mm/min. (a) initial of the hydrogel. (b) The hydrogel was stretched to 2500% of its original length. (c) Highly stretchable stress–strain behavior of the hydrogel with various stretch speed. It was demonstrated that the slower of the stretch, the larger was the elongation of the hydrogel. (d) Highly stretchable stress–strain behavior of the hydrogel with various weight ratio of PEGDA / AAm. The stretchability of hydrogel decreases with the increase of PEGDA, while the tensile stress of the hydrogel increases with the increase of PEGDA. The reason is that the PEGDA segment acted as hard domains to form the backbone of the hydrogel network, the hydrogel exhibites change of viscoelastic to elastoplastic properties with the increase of PEGDA.

**Figure S6.** The performance of a LED bulb connected by hydrogels. (a) The hydrogel worked as conductors at 20 °C. (b) The hydrogel worked as conductors at −115 °C. (c) Conventional hydrogel turned to be insulator below 0 °C. The conventional hydrogel cannot illuminate the LED performing as an insulator. (d) We also designed a complete circuit composed of a light-emitting diode (LED) bulb, increasing the elongation led to a darker light in the LED bulb. The results indicate that the electrical resistance of the hydrogel increases with the increasing stretch of the hydrogel.

**Figure S7.** Conductivity of the printed hydrogel affected by different parameters. (a) The relationship between conductivity and weight ratio of PEGDA/AAm. The conductivity of the hydrogel increased with the decrease of the content of PEGDA in the hydrogel. The reason is, the more PEGDA, the more cross-linked network, and the free ionization of LiCl will be blocked when transducing the electricity or signal by the network. (b) Comparison of hydrogels’ conductivity with different water/glycerol weight ratios. The results demonstrated that increasing the glycerol inhibits the ionization of the LiCl, resulting in a decrease of conductivity of the hydrogel. (c) Conductivity of the hydrogels with different weight concentration of LiCl. The conductivity of the hydrogel increased with the increasing concentration of LiCl, an increase of free ions from a higher concentration of LiCl in the hydrogels is the key reason for the higher conductivity of the hydrogel.

**Figure S8.** Hydrogel sensor for detection of different physiological motions. (a) The resistance of the hydrogel increased with stretching. (b)The signal of bending-unbending the wrist. (c) The signal of moving elbow. (d) The signal of rotating ankle. (e) The signal of bending-unbending knee joints. (f) When attached to the throat, the hydrogel strain sensor was able to sense human swallowing, the relative current change exhibited remarkable reproducibility when the swallowing was repeated. (g) The signal of pronouncing 26 letters in the English alphabet, the curve exhibited 26 different characteristic peaks, which was also ascribed to the high sensitivity of hydrogel sensor.

**Figure S9.** Neural interface for detecting different signals of EEG and EOG. (a) Interfacial impedance between hydrogels and the skin at different frequency, the interfacial impedance was about 12.4 kΩ, which was far below the acceptable threshold of 100 kΩ at required frequency of 10^3^ Hz. (b) The EEG signal of closing eyes. (c) The EEG signal of opening eyes. (d) The EOG nerve signal of eye balls vertical rotation. (e) The EOG nerve signal of eye balls horizontal rotation. The results validate that the hydrogel record synchronous EOG signal even better than conventional electrodes.

**Table S1. Optimized parameters for 3D printing of hydrogel using S140 printer**

| **Print parameter** | **Value** |
| --- | --- |
| LED wavelength | 405 nm |
| LED intensity | 190 mW·cm^-2^ |
| Pixel size | 10 μm |
| Exposure time | 8 s |
| Typical slice thickness | 10 μm |
| Slides per layer | 1 |

**Table S2.** Summary of results for stretchable and anti-freezing gels. Notably, the performance of the present hydrogel is much better than those of most reported gels, including the super anti-freezing property.

| **Reference** | **Technology** | **Anti -freezing materials** | **Stretchability** | **Temperature** | **Application** |
| --- | --- | --- | --- | --- | --- |
| This work | 3D printing | Gly / H2O /LiCl | 2500% | -125°C | Sensor, conductor, neural interface |
| Ref. 1 | Mold | IL | 1050% | -20°C | Electronic skin |
| Ref. 2 | Mold | H2O/EG | 960% | -55°C | Sensor |
| Ref. 3 | 3D printing | ZnCl_2_/CaCl_2_ | 120% | -70°C | Conductor |
| Ref. 4 | Mold | Betaine/Proline | -- | -40°C | Sensor |
| Ref. 5 | Mold | BzMe_3_NOH | 219% | -27.8°C | Sensor |
| Ref. 6 | Mold | H2O/Gly | 700% | -20°C | Bioelectronics, dressing |
| Ref. 7 | Mold | CaCl_2_ | 575% | -57°C | Sensor |
| Ref. 8 | Mold | H2O/Gly | -- | -30°C | Sensor, actuator |
| Ref. 9 | Mold | H2O/EG | 350% | -40°C | Sensor |
| Ref. 10 | Mold | IL | 1390% | -75°C | Triboelectric nanogenerator |
| Ref. 11 | Spinning | sodium ions | 1200% | −35 °C | Conductor |
| Ref. 12 | Mold | EG /LiCl | 229% | −80 °C | Sensor |
| Ref. 13 | Mold | IL | 5000% | −70 °C | Sensor |

**List of Movies**

**Movie S1** (.mp4 format). Flexibility and stretchablity of hydrogel at about −115 °C.

**Movie S2** (.mp4 format). Ultra-stretchable and anti-freezing properties of hydrogel.

**Movie S3** (.mp4 format). Conductive performance of hydrogel.

**Movie S4** (.mp4 format). Conductive properties of hydrogel at about −115°C.

**Movie S5** (.mp4 format). Hydrogel sensor for detection of finger motion.

**Movie S6** (.mp4 format). Tensile cyclic tests of hydrogel.

**References**

[1] Z. Liu, Y. Wang, Y. Ren, G. Jin, C. Zhang, W. Chen, F. Yan, *Mater. Horiz.* **2020**, *7*, 919.

[2] Q. Rong, W. Lei, L. Chen, Y. Yin, J. Zhou, M. Liu, *Angew. Chem. Int. Ed.* **2017**, *56*, 14159.

[3] X.-F. Zhang, X. Ma, T. Hou, K. Guo, J. Yin, Z. Wang, L. Shu, M. He, J. Yao, *Angew. Chem. Int. Ed.* **2019**, *58*, 7366.

[4] X. Sui, H. Guo, P. Chen, Y. Zhu, C. Wen, Y. Gao, J. Yang, X. Zhang, L. Zhang, *Adv. Funct. Mater.* **2019**, 1907968.

[5] Y. Wang, L. Zhang, A. Lu, *ACS Appl. Mater. Interfaces* **2019**, *11*, 41710.

[6] L. Han, K. Liu, M. Wang, K. Wang, L. Fang, H. Chen, J. Zhou, X. Lu, *Adv. Funct. Mater.* **2018**, *28*, 1704195.

[7] X. P. Morelle, W. R. Illeperuma, K. Tian, R. Bai, Z. Suo, J. J. Vlassak, *Adv. Mater.* **2018**, *30*, 1801541.

[8] Y. Jian, B. Wu, X. Le, Y. Liang, Y. Zhang, D. Zhang, L. Zhang, W. Lu, J. Zhang, T. Chen, *Research* **2019**, *2019*, 2384347.

[9] H. Liao, X. Guo, P. Wan, G. Yu, *Adv. Funct. Mater.* **2019**, *29*, 1904507.

[10] Y. Ren, J. Guo, Z. Liu, Z. Sun, Y. Wu, L. Liu, F. Yan, *Sci. Adv.* **2019**, *5*, eaax0648.

[11] X. Zhao, F. Chen, Y. Li, H. Lu, N. Zhang, M. Ma, *Nat. Commun.* **2018**, *9*, 3579.

[12] D. Lou, C. Wang, Z. He, X. Sun, J. Luo, J. Li, *Chem. Commun.* **2019**, *55*, 8422.

[13] Z. Cao, H. Liu, L. Jiang, *Mater. Horiz.* **2020**, *7*, 912.
